# Biomarker Discovery for Cytochrome P450 1A2 Activity Assessment in Rats, Based on Metabolomics

**DOI:** 10.3390/metabo9040077

**Published:** 2019-04-18

**Authors:** Xiao Pu, Yiqiao Gao, Ruiting Li, Wei Li, Yuan Tian, Zunjian Zhang, Fengguo Xu

**Affiliations:** 1 Key Laboratory of Drug Quality Control and Pharmacovigilance (Ministry of Education), State Key Laboratory of Natural Medicine, China Pharmaceutical University, Nanjing 210009, China; puxiao54321@163.com (X.P.); gaoyiqiao92@126.com (Y.G.); 15051853218@163.com (R.L.); 15250958887@163.com (W.L.); 1020041334@cpu.edu.cn (Y.T.); 2 Jiangsu Key Laboratory of Drug Screening, China Pharmaceutical University, Nanjing 210009, China

**Keywords:** CYP1A2, endogenous biomarkers, metabolomics, branched-chain amino acids, phenylalanine, tyrosine

## Abstract

Cytochrome P450 1A2 (CYP1A2) is one of the major CYP450 enzymes (CYPs) in the liver, and participates in the biotransformation of various xenobiotics and endogenous signaling molecules. The expression and activity of CYP1A2 show large individual differences, due to genetic and environmental factors. In order to discover non-invasive serum biomarkers associated with hepatic CYP1A2, mass spectrometry-based, untargeted metabolomics were first conducted, in order to dissect the metabolic differences in the serum and liver between control rats and β-naphthoflavone (an inducer of CYP1A2)-treated rats. Real-time reverse transcription polymerase chain reaction and pharmacokinetic analysis of phenacetin and paracetamol were performed, in order to determine the changes of mRNA levels and activity of CYP1A2 in these two groups, respectively. Branched-chain amino acids phenylalanine and tyrosine were ultimately focalized, as they were detected in both the serum and liver with the same trends. These findings were further confirmed by absolute quantification via a liquid chromatography–tandem mass spectrometry (LC-MS/MS)-based targeted metabolomics approach. Furthermore, the ratio of phenylalanine to tyrosine concentration was also found to be highly correlated with CYP1A2 activity and gene expression. This study demonstrates that metabolomics can be a potentially useful tool for biomarker discovery associated with CYPs. Our findings contribute to explaining interindividual variations in CYP1A2-mediated drug metabolism.

## 1. Introduction

As one of the most important enzyme systems for drug metabolism in the human body, CYP450 enzymes (CYPs) play a crucial role in the metabolism of xenobiotics and endogenous substrates [1]. CYP1A2 is one of the major CYPs in the human liver, and accounts for about 13% of the total CYP protein content [2]. CYP1A2 is responsible for the oxidative metabolism of 8–10% of the marketed drugs [3] and the metabolic activation of environmental carcinogens, such as polycyclic aromatic hydrocarbons and heterocyclic amines [4,5]. In addition, CYP1A2 also contributes to the biotransformation of various endogenous compounds including polyunsaturated fatty acids, melatonin, estrone, estradiol, etc. [6]. The expression and activity of CYP1A2 varies 40–130 times between individuals [6,7] due to constitutional and environmental factors, such as gene polymorphism, gender, age, ethnicity, smoking status, and diet [8,9,10]. The wide interindividual variability in CYP1A2 activity is a crucial reason for variations in drug response, and is associated with drug efficacy and toxicity. Meanwhile, extensive studies have shown that the risk of lung cancer [11,12], bladder cancer [13], gastric cancer [14], and colorectal cancer [15,16] is associated with CYP1A2 gene polymorphism. Hence, phenotyping CYP1A2 activity has potential clinical importance for precision medicine.

Traditionally, pharmacokinetics (PK) of specific probe drugs, such as phenacetin, theophylline, and caffeine, are used to assess CYP1A2 activity in vivo [17]. However, probe drug assays require the intake of non-therapeutic test drugs, multiple blood samplings, and analysis of both parent drugs and their metabolites, which makes the process too invasive, complicated, inconvenient for widespread use, and lacking in desired safety and tolerability [18]. Thus, identifying better probe substrates that can act as noninvasive biomarkers of CYP1A2 is of great necessity. Up to now, several endogenous biomarkers of CYPs have been reported. For example, plasma 4β-hydroxycholesterol levels and urinary 6β-hydroxycortisol-to-cortisol ratio have been used successfully to reflect CYP3A activity [19,20]. However, up to now, no endogenous biomarkers for CYP1A2 have been identified. 

Metabolomics aims to gather as much information about low-molecule metabolites in biological systems as possible, and has been demonstrated to be a promising and powerful approach to elucidate the metabolizing properties and physiological functions of a CYP enzyme [21]. More sensitive and reliable biomarkers of CYPs could be identified using a metabolomic approach, as it reflects comprehensive metabolic changes in response to genetic alterations and environmental interventions [22,23].

The goal of the present study is to discover endogenous serum biomarkers that can indicate hepatic CYP1A2 activity. The compound β-Naphthoflavone was used as a tool compound to develop a mechanism-based induction model of CYP1A2. The changes in mRNA levels and activity of CYP1A2 were determined by a real-time reverse transcription polymerase chain reaction (RT-PCR) and PK analysis of phenacetin and paracetamol. An integrated gas (GC-MS) and liquid chromatography mass spectrometry (LC-MS)-based, untargeted metabolomic approach was applied to identify potential biomarkers that were highly correlated with CYP1A2 induction. These biomarkers were further confirmed by a liquid chromatography–tandem mass spectrometry (LC–MS/MS)-based targeted metabolomics.

## 2. Materials and Methods

### 2.1. Chemicals and Reagents

β-Naphthoflavone (BNF, ≥99.0%); phenylalanine (Phe, 99.0%); tyrosine (Tyr, ≥99.0%); branched-chain amino acids (BCAAs), including valine (Val, ≥99.5%), leucine (Leu, ≥99.5%), and isoleucine (Ile, ≥ 99.5%); ^13^C_1_-leucine (internal standard (IS-2), 99.0%); *O*-Methoxyamine hydrochloride (MOX); *N*-methyl-*N*-trifluoroacetamide (MSTFA); and pyridine were purchased from Sigma-Aldrich (St. Louis, MO, United States). Phenacetin (≥98.0%), paracetamol (≥99.5%), and corn oil were purchased from Aladdin Biochemical Technology Co., Ltd. (Shanghai, China). Pseudoephedrine hydrochloride (internal standard (IS-1), ≥ 98.0%) was obtained from the National Institutes for Food and Drug Control (Beijing, China). Sodium carboxymethyl cellulose (CMC-Na) of analytical grade was obtained from Sinopharm Chemical Reagent Co., Ltd. (Shanghai, China). RNAiso Plus and a PrimeScript RT reagent kit were purchased from Takara Medical CO., Ltd. (Kyoto, Japan). The LightCycler 480 SYBY Green I Master was purchased from Roche (Mannheim, Germany). LC-MS grade methanol, acetonitrile, and ethyl acetate were purchased from Merck (Darmstadt, Germany). High-performance liquid chromatography (HPLC)-grade formic acid was purchased from ROE Scientific Inc. (Newark, NJ, USA). Sodium bicarbonate anhydrous (NaHCO_3_) of analytical grade was obtained from Nanjing Chemical Reagent Co., Ltd. (Nanjing, China). Ultrapure deionized water was prepared by a Milli-Q system (Millipore, Bedford, MA, USA). 

### 2.2. Animal Experiments and Sample Collection

All animal experiments were performed in accordance with the Guide for the Care and Use of Laboratory Animals and were approved by the Animal Ethics Committee of China Pharmaceutical University (No. 1621010273). Sixteen male Sprague–Dawley rats (specific pathogen-free grade, 180–200 g, 6–7 weeks of age) were purchased from the Sino-British SIPPR/BK Lab Animal Ltd. (Shanghai, China). All animals were housed in routine laboratory conditions (temperature: 22–26 °C; relative humidity: 50 ± 5%; light–dark cycle: 12/12 h), with free access to water and food. After a week of acclimatization, rats were randomly divided into a control group (C; *n* = 8) and a β-naphthoflavone (BNF)-treated group (*n* = 8). The BNF group animals were intraperitoneally (i.p.) injected with 80 mg/kg β-naphthoflavone dissolved in corn oil once a day for three consecutive days (day 1–3) [24], while animals in the C group received a corresponding administration of the vehicle (corn oil, 10 mL/kg). At day 4, 300 μL of orbital venous blood of each rat was collected into clean tubes, followed by coagulation (1 h) and centrifugation (4 °C, 5000 rpm, 10 min) (Eppendorf 5430R, Germany). Subsequently, all rats were intragastrically administered with phenacetin suspended in 0.5% (*w*/*v*) CMC-Na aqueous solution, at a dose of 15 mg/kg [25,26]. Blood samples (250–300 μL) from the fossa orbital vein were collected into heparinized centrifuge tubes pre-dose (0 h) and 0.083, 0.25, 0.5, 1, 2, 4, 6, 8, and 12 h after administration; the samples were then immediately separated by centrifugation at 5000 rpm for 10 min at 4 °C. The obtained serum and plasma samples were then aliquoted and stored at −80 °C prior to metabolomic and PK analysis, respectively. Rats were sacrificed after the final 12 h post-dose, and each liver sample was removed, rinsed in ice-cold saline, quickly frozen in liquid nitrogen, and stored at −80 °C until mRNA determination or metabolomic analysis. The dosage of BNF and phenacetin and the experimental period were determined according to the existing literature and the results of our pre-experiments. The overall animal experimental scheme is shown in Supplementary Figure S1.

### 2.3. Pharmacokinetic Studies

Phenacetin and its metabolite paracetamol were simultaneously determined by an LC-MS-validated method. A plasma sample was analyzed on a ZORBAX Eclipse XDB-C18 (150 × 2.1 mm, 3.5 μm) (Agilent Technologies, Santa Clara, CA, United States). Mass spectrometry (MS) analysis was performed on a LC-MS2010 mass spectrometer equipped with an electron spray ionization (ESI) source (Shimadzu, Tokyo, Japan). Sample preparation and analytical methods are shown in Supplementary Methods 1.1. PK parameters were calculated by DAS 3.2 (Mathematical Pharmacology Professional Committee of China, Shanghai, China) and compared between groups using a Student’s *t*-test using PASW Statistics 19 (SPSS Inc., Chicago, IL, USA).

### 2.4. Determination of Cytochrome P450 1A2 mRNA Levels by Reverse Transcription Polymerase Chain Reaction

Approximately 50 mg of hepatic tissues were homogenized, and the total RNA was isolated by RNAiso plus (Kyoto, Japan), according to the manufacturer’s instructions. The RNA concentration was determined at 260 nm, and the quality was assessed by measuring the absorbance ratio of 260/280 nm using a Tecan Infinite 200 Pro (Tecan Group Ltd., Mannedorf, Switzerland). Reverse transcription was performed using a PrimeScript RT reagent kit (Kyoto, Japan), according to the supplier’s protocols. The polymerase chain reaction was conducted on a LightCycler 96 Real-Time PCR System (Roche, Mannheim, Germany) using a LightCycler 480 SYBY Green I Master (Mannheim, Germany). The house-keeping gene, *β-actin*, was used as an internal reference standard. The relative expression of *CYP1a2* was calculated using the 2^-ΔΔ*Ct*^ method [27]. Data were expressed as the fold change of the target genes relative to control. The primer pairs used in RT-PCR are summarized in Table 1.

### 2.5. Untargeted Metabolomics Analysis

Serum and liver sample pretreatments, GC-MS and LC-MS analysis, data preprocessing, and metabolite identification were all based on our previous studies [28,29,30] (as shown in Supplementary Methods 1.2). After metabolic information collection and data preprocessing, the resulting matrix was imported to SIMCA-P (version 13.0, Umetrics, Sweden) for principal component analysis (PCA) and orthogonal partial least-squares-discriminant analysis (OPLS-DA). PCA was used to get a preliminary overview of grouping trends, while OPLS-DA was employed to identify the potential biomarkers between the control and BNF groups. The parameter *Q*^2^ of OPLS-DA represents the predictive ability of the model. Differential variables correlated with CYP1A2 induction were screened out as follows: first, variable importance in the projection (VIP) value, which represents the contribution for grouping, should be greater than 1.0. Second, in order to reduce the probability of false positives, an adjusted *p* value of the nonparametric Mann-Whitney U test (PASW Statistics 19, SPSS Inc., Chicago, United States) where the Benjamini–Hochberg false discovery rate (FDR) correction should be lower than 0.05 [31]. Third, the value of the area under the receiver operating characteristic (AUC-ROC) was calculated using PASW Statistics 19 (SPSS Inc., Chicago, IL, United States), and the variables were discarded when AUC-ROC ≤ 0.8. AUC-ROC is an evaluation parameter for classification performance and the classification performance can be considered excellent when AUC-ROC > 0.9 [30]. Fourth, after Spearman correlation analysis (PASW Statistics 19, SPSS Inc., Chicago, United States), variables should have significant correlations with both the mean metabolic ratio of paracetamol:phenacetin and the mRNA level of CYP1A2. The value of the metabolic ratio reflects the activity of CYP1A2, and a higher value represents greater activity. CYP1A2 gene expression was calculated using the 2^−Δ*Ct*^ method. Differential variables were screened out according to the above screening criteria, and then prepared for further identification. Identified differential metabolites were ultimately focalized if they were detected from both serum and liver and had the same change trends in the two biological samples. Metabolites heatmaps were performed by MultiExperiment View (Version 4.9.0).

### 2.6. Data Quality Evaluation in Untargeted Metabolomics Analysis

Quality control samples (QCs) were made by pooling equal aliquots of each serum or liver homogenate sample. After mixing absolutely, the QCs were analyzed consistently with real samples. Before the batch analysis, 10 QCs were first tested to stabilize the analytical system, and the acquired data was removed before data processing. All QCs were inserted randomly through the analytical batch to monitor the robustness of sample preparation and the stability of instrument analysis. All serum and liver samples were randomly analyzed during the whole instrumental analysis to avoid inter-batch differences [29]. In order to evaluate model overfitting, permutation tests (200 times) were performed in the partial least-squares–discriminant analysis (PLS-DA) model, with the same number of components of the corresponding OPLS-DA model [32].

### 2.7. Targeted Metabolomic Analysis of Branched-Chain Amino Acids, Phenylalanine, and Tyrosine

A simple and rapid analytical method was developed for simultaneous quantification of branched-chain amino acids (BCAAs), including valine (Val), leucine (Leu), isoleucine (Ile), phenylalanine (Phe), and tyrosine (Tyr), based on our previous study with little modification [33]. Sample analysis was performed on an Agilent ZORBAX SB-C18 column (100 × 3 mm, 3.5 μm; Agilent, MA, USA), and detected by a LC/MS 8040 triple-quadrupole mass spectrometer with an ESI source (Shimadzu, Kyoto, Japan). The details of sample preparation and analytical methods are provided in Supplementary Methods 1.3. Full method validation was carried out in terms of linearity, limit of detection, limit of quantification, accuracy, precision, extraction recovery, matrix effect, and stability, according to the U.S. Food and Drug Administration document and other related guidelines [34,35].

## 3. Results

### 3.1. β-Naphthoflavone-Induced Cytochrome P450 1A2 Activity and mRNA Expression

**Figure 1:**
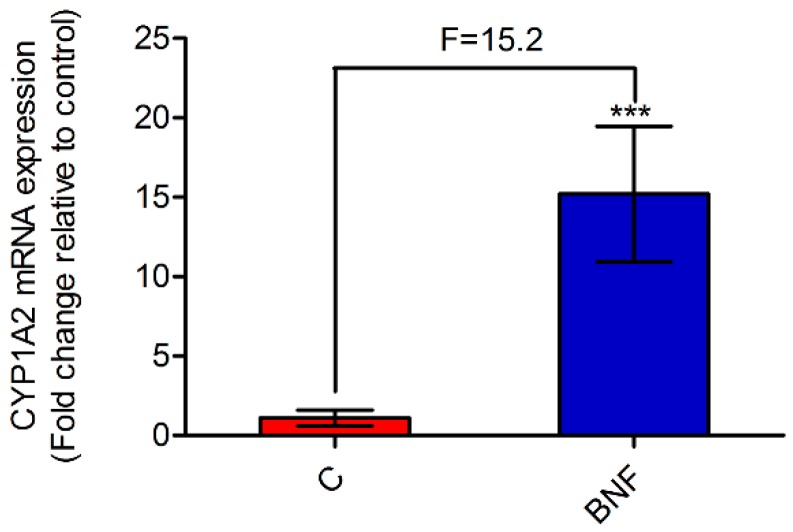
The relative expression of cytochrome P450 1A2 (CYP1A2) mRNA after β-naphthoflavone administration. The mRNA levels were normalized by *β-actin* expression, and expressed as the fold change relative to control. Data are expressed as mean ± SD, and *n* = 8 for each group. Unpaired Student’s *t*-test. *** *p* < 0.001. C: control group; BNF: β-naphthoflavone treatment group; F: fold change.

A developed and validated LC-MS method was performed to determine the plasma concentration of phenacetin and its metabolite, paracetamol. PK parameters of phenacetin were used as the gold standard for assessing CYP1A2 activity [18]. Representative LC-MS chromatograms of phenacetin, paracetamol, internal standard (IS-1, pseudoephedrine hydrochloride, and concentration–time curves of phenacetin and paracetamol are all shown in Supplementary Figure S2. As can be seen from Table 2, the AUC_(0-12h)_ and C_max_ of phenacetin were decreased significantly in the BNF group compared with the control group. Besides, the mean metabolic ratio of paracetamol:phenacetin, which strongly indicates CYP1A2 activity in the metabolic pathway, increased from 0.77 to 34.58. The results of PK studies indicated that β-naphthoflavone potently induced rat hepatic CYP1A2 activity, and evidently accelerated the metabolism of phenacetin. Furthermore, Figure 1 shows that the CYP1A2 mRNA level increased 15.2-fold after β-naphthoflavone administration, which was in accordance with the PK results. From the above, the CYP1A2 induction model was successfully established by continuous administration of β-naphthoflavone (80 mg/kg/day, 3 days, i.p.), based on the results of PK and RT-PCR analyses.

### 3.2. Data Quality Evaluation in Untargeted Metabolomics

As shown in Supplementary Figure S3, for both the serum and liver samples analyzed by GC-MS and LC-MS, QCs were clustered tightly in PCA score plots, indicating good reproducibility of the data. The retention time shift was less than 0.1 min, and the relative standard deviation (RSD) values of all QCs peaks were below 30%, which demonstrates that our analytical method is stable and reliable [36]. Moreover, the permutation test (200 times) results (Supplementary Figure S4D–F,J–L) showed negative *Q*^2^-intercept values and lower permuted *R*^2^-values compared with the original *R*^2^-value, which confirms that these OPLS-DA models were not overfitting [28].

### 3.3. Biomarker Screening Based on Untargeted Metabolomic Analysis

**Figure 2:**
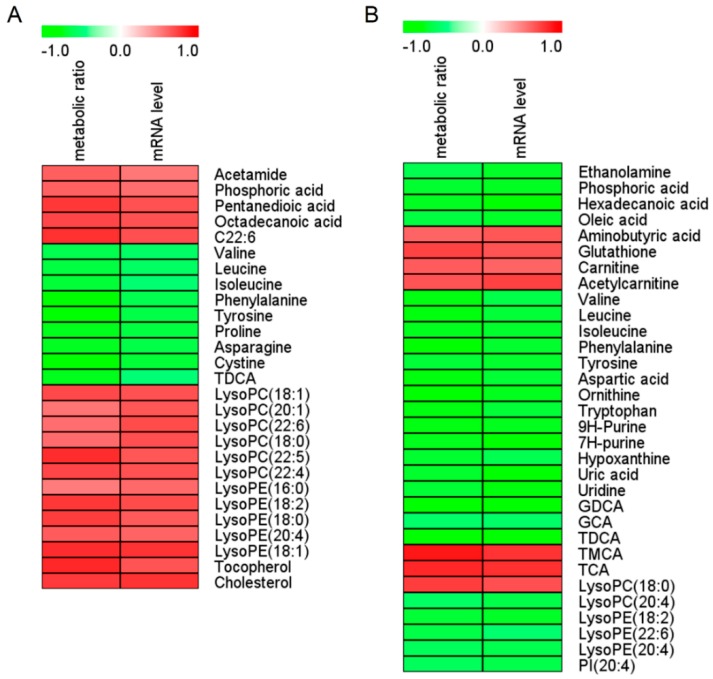
Spearman correlation analysis of serum (**A**) and liver (**B**) marker metabolites induced by β-naphthoflavone as well as the metabolic ratio and mRNA level of CYP1A2. Green squares indicate significant negative correlations (−0.51 to −0.90 for serum, −0.53 to −0.89 for liver; *p* < 0.05), white squares indicate non-applicable correlations, and red squares indicate significant positive correlations (0.52 to 0.83 for serum, 0.61 to 0.89 for liver; *p* < 0.05). The metabolic ratio is AUC_0-12 h_ of paracetamol/AUC_0–12h_ of phenacetin. AUC_0–12h_ is the area under the plasma concentration–time curve from 0 to 12 h. The value of the metabolic ratio reflects CYP1A2 activity, and a higher value represents greater activity. The CYP1A2 mRNA level was calculated using the 2^-Δ*Ct*^ method. *Ct*: cycle threshold; Δ*Ct* = *Ct* of *CYP1a2* - *Ct* of *β-actin*; C22:6: cis-4,7,10,13,16,19-docosahexaenoic acid; GCA: glycocholic acid; GDCA: glycodeoxycholic acid; TDCA: taurodeoxycholic acid: LysoPC, lysophosphatidylcholine; LysoPE: lysophosphatidylethanolamine; PI: phosphoinositol; TCA: taurocholic acid; TMCA: tauromuricholic acid.

In order to find differential metabolites between the C and BNF groups, untargeted metabolomic analysis of the serum and liver samples was performed. PCA score plots were first applied, to reveal the overall differences between groups. As can be seen from Supplementary Figure S3, the BNF group was obviously separated from the control group. OPLS-DA score plots were performed to identify the differential metabolites between the C and BNF groups. As shown in Supplementary Figure S4A–C,G–I, the C and BNF groups were completely separated in OPLS-DA score plots, with a prediction power score of *Q*^2^ > 0.5. According to the biomarker screening criteria, 27 differential metabolites in the serum (Figure 2A and Supplementary Tables S4 and S5) and 32 differential metabolites in the liver (Figure 2B and Supplementary Tables S6 and S7) that correlated with CYP1A2 activity and gene expression were screened out and identified, respectively. In order to discover serum biomarkers that can indicate hepatic CYP1A2 activity, BCAAs (Val, Leu, and Ile), Phe, Tyr, TDCA and LysoPC(18:0) were ultimately focalized, as all of them were detected from both serum and liver and had the same change trends. As shown in Table 3, β-naphthoflavone administration caused a reduction in BCAAs, Phe, Tyr, and TDCA, but an increase in LysoPC(18:0) compared with group C, based on area normalization data of untargeted metabolomics. Meanwhile, all of these differential metabolites were strongly correlated with both CYP1A2 activity and gene expression, and had low *p*FDR values and high AUC-ROC values. ROC curves of seven focalized metabolites were shown in Supplementary Figure S5.

### 3.4. Targeted Quantification of Val, Leu, Ile, Phe and Tyr

**Figure 3:**
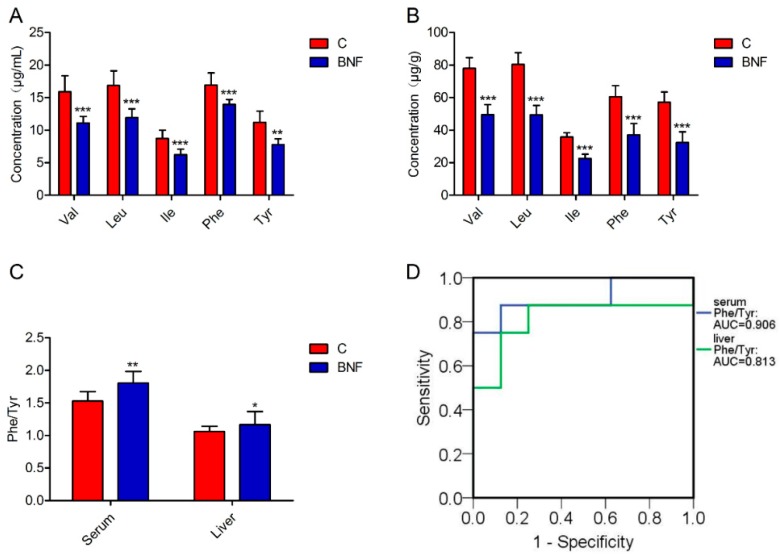
Targeted metabolomic analysis of the focalized biomarkers. (**A**) Concentration of Val, Leu, Ile, Phe, and Tyr in serum. (**B**) Concentration of Val, Leu, Ile, Phe, and Tyr in liver. (**C**) The ratio of Phe to Tyr concentration in the serum and liver. (**D**) ROC curves of Phe/Tyr in serum and liver. Data are presented as mean ± SD, and *n* = 8 for each group. Nonparametric Mann–Whitney U test. * *p* < 0.05; ** *p* < 0.01; *** *p* < 0.001; C: control group; BNF: β-naphthoflavone treatment group; Val: valine; Leu, leucine: Ile, isoleucine; Phe: phenylalanine; Tyr: tyrosine; and Phe/Tyr, the ratio of Phe to Tyr concentration.

In order to further confirm our untargeted metabolomics findings, serum and liver metabolomic profiles of Val, Leu, Ile, Phe, and Tyr were absolutely quantified by a fully validated LC-MS/MS-based targeted metabolomic method. A representative multiple reaction monitoring (MRM) chromatogram of each analyte in a serum of normal rats is shown in Supplementary Figure S6. Consistent with untargeted results, the concentration of each analyte shows a statistically significant difference between the control and BNF groups, revealing decreased content of Val, Leu, Ile, Phe, and Tyr in both serums (Figure 3A and Supplementary Table S8) and lthe iver (Figure 3B and Supplementary Table S8). For each focalized metabolite, AUC-ROC was calculated (PASW Statistics 19, SPSS Inc., Chicago, United States) based on quantitative results. AUC-ROC value of Val, Leu, Ile, Phe, and Tyr in the serum was 0.984, 1.000, 1.000, 1.000, and 0.969, respectively, while those in the liver were all 1.000, indicating that Val, Leu, Ile, Phe, and Tyr were satisfactory indicators of the individual differences of CYP1A2 activity in rats. In addition, a targeted analysis of Phe and Tyr showed that the ratio of Phe to Tyr concentration (Phe/Tyr) was significantly increased in β-naphthoflavone treated rats (Figure 3C and Supplementary Table S9), with high AUC-ROC values (Figure 3D). Moreover, correlation analysis showed that serum Phe/Tyr was also highly correlated with CYP1A2 activity and gene expression (*p* < 0.05) (data not shown).

## 4. Discussion

CYP1A2 shows remarkable conservation among species, with an identity in human >80% that of rats [37], so a CYP1A2-mediated signaling pathway and metabolic profile in human beings could be studied by studying rats. In the present study, rats were picked as animal model to screen biomarkers of CYP1A2 activity. Compared to other research subjects, such as knockout mice [22] and healthy humans [23], the biomarkers screening method based on selective inducer induced-animals is more convenient and economical. In addition, only male rats were included in the study, in order to avoid interference from estrogen on CYP1A2 activity [38,39]. The rat-based biomarkers would be a promising basis and reference for future development and application in the human body.

In order to discover reliable biomarkers of CYP1A2, Spearman correlation analysis was performed between altered metabolites with the mean metabolic ratio of paracetamol:phenacetin and mRNA level of CYP1A2. PK parameters of phenacetin were used as the gold standard for determining CYP1A2 activity in the current study, due to the availability of the parent substrate and metabolite, and the simple and fast HPLC-MS detection assay with high sensitivity. Although phenacetin is also a CYP2C6 substrate in rats [40], as far as we know, the effect of β-naphthoflavone on CYP2C6 activity has not been reported and needs further study. Hence, for the time being, we can conclude that the changes in PK parameters of phenacetin were caused by changes in CYP1A2 activity, rather than CYP2C6. Moreover, Spearman correlation analysis between differential metabolites and the CYP1A2 mRNA level also avoided interference from CYP2C6 to a certain extent.

β-Naphthoflavone potently induces CYP1A2 expression through the aromatic hydrocarbon receptor (AHR)-mediated signaling pathway [41], and is widely used as a positive control to characterize CYP1A2 induction of a drug or herb compound [24,42]. In the present study, β-naphthoflavone was used as a tool compound to develop a mechanism-based induction model of CYP1A2. Consistent with previous reports [42,43], we found that β-naphthoflavone also highly induced mRNA expression of CYP1A1 (Supplementary Figure S7). In addition, the extent of induction of CYP1A1 (1270.3-fold) was remarkably higher than that of CYP1A2 (15.2-fold). The possibility that the changes in metabolic profiles were partially caused by β-naphthoflavone as an active compound itself, as well as the induction of CYP1A1 and other AHR-mediated enzymes like CYP1B1 (not measured in the current study), cannot be excluded. However, Spearman correlation analysis was performed between altered differential variables with both the mean metabolic ratio of paracetamol:phenacetin and the mRNA level of CYP1A2, and the variables were discarded when the correlations were not significant (*p* > 0.05). Hence, we could conclude that the screened and identified metabolites were strongly correlated with both CYP1A2 activity and gene expression.

GC-MS analysis mainly focuses on strong polar metabolites, such as amino acids, short chain fatty acids, and carbohydrates, while LC-MS analysis mainly focuses on weak polar metabolites, such as phospholipids, long chain fatty acids, and bile acids [30]. Hence, an integrated GC-MS and LC-MS-based, untargeted metabolomic approach was applied, in order to more comprehensively characterize the changes of metabolic profiles in rats. After β-naphthoflavone treatment, 27 and 32 differential metabolites correlated with CYP1A2 induction in the serum and liver were screened out and identified. Since the sampling of the liver was more invasive, we focused on serum biomarkers that were also detected in liver, and had the same change trends as with the liver samples. Ultimately, Val, Leu, Ile, Phe, Tyr, TDCA, and LysoPC(18:0) were focused for the next research. Due to the relative quantification of metabolites and complicated data processing, the untargeted analysis is often less accurate. Hence, the absolute concentrations of the focalized biomarkers were quantitatively determined using an LC-MS/MS-based, targeted metabolomic approach. Here in this paper, amino acids, including Val, Leu, Ile, Phe, and Tyr were simultaneously quantified to confirm the results of untargeted assays. As a result, AUC-ROC values of all the focalized serum biomarkers in targeted assays were higher than those in untargeted assays, which further indicates the necessary of targeted determination in untargeted metabolomic studies.

BCAAs are essential amino acids that must be obtained from diet. Catabolism of BCAAs is initiated by reversible transamination to form branched-chain ketoacids (BCKAs). Then, BCKAs are irreversibly decarboxylated by the branched-chain ketoacid dehydrogenase complex, and are eventually degraded into succinyl-CoA and acetyl-CoA, to fuel the tricarboxylic acid cycle [33]. There is a growing body of literature to suggest that BCAAs play a key role in cancer development [44,45], and that the serum level of BCAAs can be used to indicate the status of various diseases, such as cardiovascular diseases [46] and diabetes mellitus [47]. Phe is one of the essential amino acids, whereas Tyr is referred to as semi-essential. Phe is irreversibly hydroxylated by phenylalanine hydroxylase to form Tyr, and Tyr is further hydroxylated by tyrosine hydroxylase to produce dopa, the precursor of dopamine, norepinephrine and epinephrine [48]. In recent years, it has become evident that Phe/Tyr is a potential indicator of inflammatory diseases like cancer [49], sepsis [50], and chronic kidney failure [51], as well as Parkinson’s disease [48] and depression [52]. After β-naphthoflavone treatment, we found that BCAA, Phe, and Tyr levels were significantly decreased, while Phe/Tyr was significantly increased; all of the amino acids were highly correlated with both CYP1A2 activity and gene expression. However, the mechanism of how CYP1A2 acts in BCAAs and the Phe and Tyr metabolism is unknown, and needs further investigation.

Although CYP1A2 shows strong conservation between human and rats, the extent to which CYP1A2 expression and activity changes are caused by gene polymorphism, environmental implication, and disease status in a normal population, cannot be fully replicated by artificial intervention using an inducer or inhibitor. Moreover, the species differences of CYP1A2-mediated metabolism between rats and human should be concerned. In the next step, we plan to predict human hepatic CYP1A2 activity by measuring the identified serum biomarkers, to further confirm our findings. In addition, more exploratory studies needed to be carried out to investigate whether age, gender, and ethnicity affect the serum metabolic signature of hepatic CYP1A2. Although it is unclear whether our findings could be applied clinically, our study provided methods and ideas to develop noninvasive serum biomarkers that can be used to monitor hepatic CYP activity without the necessity of drug treatment or serial blood sampling for PK analysis.

## 5. Conclusions

In conclusion, in the present study, an integrated GC-MS and LC-MS untargeted metabolomic approach was performed to discover endogenous biomarkers of CYP1A2, based on β-naphthoflavone-induced rat models. BCAAs, Phe, Tyr, and Phe/Tyr were first proposed as serum biomarkers for hepatic CYP1A2 activity assessment. Our methods and findings provide a noninvasive means of CYP1A2 phenotyping and improve current drug therapy mediated by CYP1A2 metabolism.

## Figures and Tables

**Table 1:** Sequences of the primers of *CYP1a2* and *β-actin.*

Gene	Sequence (5′-3′)	Product Size (bp)
*CYP1a2*	Forward: GCCATCTTCTGGAGCATTTTG	208
	Reverse: TGTCCCTCGTTGTGCTGTG	
*β-actin*	Forward: GGAGATTACTGCCCTGGCTCCTA	150
	Reverse: GACTCATCGTACTCCTGCTTGCTG	

**Table 2:** Pharmacokinetic (PK) parameters after a single intragastric administration of phenacetin.

Parameter	C Group	BNF Group
AUC_0-12h_ (μg/L·h)	3290.08 ± 302.66	105.63 ± 66.41 ***
C_max_ (μg/L)	4469.16 ± 331.91	313.84 ± 229.00 ***
T_1/2_ (h)	0.47 ± 0.37	0.32 ± 0.19
Metabolic ratio ^a^	0.77 ± 0.11	34.58 ± 22.02 **

Data are expressed as mean ± standard deviation (SD), and *n* = 8 for each group. Unpaired Student’s *t*-test. ** *p* < 0.01; *** *p* < 0.001. C: control group; BNF: β-naphthoflavone treatment group; AUC_0-12h_: area under the plasma concentration–time curve, from 0 to 12 h; C_max_: maximum plasma concentration; T_1/2_: terminal half-life; ^a^ AUC_0-12h_ of paracetamol/AUC_0-12h_ of phenacetin.

**Table 3:** List of seven differential metabolites, focalized based on untargeted metabolomic analysis.

NO.	Metabolites	VIP Value	*p*FDR	r Value (Metabolic Ratio) ^a^	r Value (mRNA level) ^b^	AUC-ROC	Change Trend ^c^	Detected From	Detected By
1	Valine	1.21	0.012	−0.63	−0.56	0.89	↓	serum	GC-MS
		1.57	0.000	−0.75	−0.64	1.00	↓	liver	GC-MS
2	Leucine	1.27	0.006	−0.66	−0.57	0.92	↓	serum	GC-MS
		1.60	0.000	−0.76	−0.68	1.00	↓	liver	GC-MS
3	Isoleucine	1.12	0.009	−0.68	−0.52	0.91	↓	serum	GC-MS
		1.60	0.000	−0.74	−0.71	1.00	↓	liver	GC-MS
4	Phenylalanine	1.07	0.007	−0.77	−0.62	0.94	↓	serum	LC-MS
		1.52	0.000	−0.78	−0.71	1.00	↓	liver	GC-MS
5	Tyrosine	1.26	0.006	−0.79	−0.65	0.92	↓	serum	GC-MS
		1.55	0.000	−0.68	−0.71	1.00	↓	liver	GC-MS
6	TDCA	1.98	0.028	−0.74	−0.51	0.84	↓	serum	LC-MS
		4.56	0.000	−0.77	−0.77	0.98	↓	liver	LC-MS
7	LysoPC(18:0)	1.58	0.005	0.58	0.69	0.94	↑	serum	LC-MS
		1.19	0.001	0.75	0.69	0.97	↑	liver	LC-MS

^a^ Correlation coefficients of Spearman correlation analysis between differential metabolites and metabolic ratio. ^b^ Correlation coefficients of Spearman correlation analysis between differential metabolites and mRNA level of CYP1A2. ^c^ Change trends of differential metabolites based on area normalization data in untargeted metabolomics. ↓ decreasing change trend after β-naphthoflavone administration. ↑ increasing change trend after β-naphthoflavone administration. The value of the metabolic ratio reflects the activity of CYP1A2, and a higher value represents greater activity. CYP1A2 mRNA expression was calculated using the 2^−Δ*Ct*^ method. *Ct*: cycle threshold. Δ*Ct* = *Ct* of *CYP1a2* - *Ct* of *β-actin*; LysoPC(18:0): Lysophosphatidylcholines(18:0); TDCA: taurodeoxycholic acid.

## Supplementary Materials

The following are available online at https://www.mdpi.com/2218-1989/9/4/77/s1, Supplementary Methods 1.1–1.3, Figures S1–S7, Tables S1–S9.
