# Supplementary material for: Biomarker Discovery for Cytochrome P450 1A2 Activity Assessment in Rats, Based on Metabolomics
Source: Metabolites. 2019 Apr 18;9(4):77. doi: 10.3390/metabo9040077 (PMC6523085; doi:10.3390/metabo9040077)
Supplement: Supplementary file 1 [file metabolites-09-00077-s001.pdf]

metabolites

Biomarker Discovery for Cytochrome P450 1A2 Activity Assessment in Rats, Based on Metabolomics

Xiao Pu [1,2], Yiqiao Gao [1,2], Ruiting Li [1,2], Wei Li [1,2], Yuan Tian [1,2], Zunjian Zhang [1,2,*] and Fengguo Xu [1,2,*]

[1] Key Laboratory of Drug Quality Control and Pharmacovigilance (Ministry of Education), State Key Laboratory of Natural Medicine, China Pharmaceutical University, Nanjing 210009, China; puxiao54321@163.com (X.P.); gaoyiqiao92@126.com (Y.G.); 15051853218@163.com (R.L.); 15250958887@163.com (W.L.); 1020041334@cpu.edu.cn (Y.T.)

[2] Jiangsu Key Laboratory of Drug Screening, China Pharmaceutical University, Nanjing 210009, China

* Correspondence: zunjianzhangcpu@hotmail.com (Z.Z.); fengguoxu@gmail.com (F.X.); Tel.: +86-25-8327-1454 (Z.Z.); +86-25-8327-1021 (F.X.)

Received: 1 April 2019; Accepted: 15 April 2019; Published: 18 April 2019

metabolites

Table of contents

1. Methods....3

1.1 Quantification of phenacetin and paracetamol....3

1.2 Untargeted metabolomics analysis....3

1.3 Quantification of BCAAs, Phe and Tyr....6

References....6

2. Figures....8

Figure S1....8

Figure S2....9

Figure S3....10

Figure S4....11

Figure S5....12

Figure S6....13

Figure S7....14

3. Tables....15

Table S1....15

Table S3....15

Table S4....16

Table S5....17

Table S6....18

Table S7....19

Table S8....21

Table S9....21

1. Methods

1.1 Quantification of phenacetin and paracetamol

For sample preparation, an aliquot of 80 μL plasma sample was pipetted into a 1.5 mL Eppendorf tube, followed by addition of 10 μL internal standard working solution (1 μg/mL pseudoephedrine hydrochloride, IS-1), 10 μL methanol, and 50 μL saturated $NaHCO_3$. Analytes were extracted with 800 μL ethyl acetate by vortexing for 3 min, and then centrifuged at 8000 rpm for 10 min at 4 °C. A total of 750 μL aliquot of the organic layer was evaporated to dryness under a gentle stream of nitrogen at 37 °C, and the resulting residue was reconstituted in 80 μL methanol-water (20:80, v/v). After 10 min of centrifugation (14000 rpm, 4 °C), 5 μL of the supernatant was injected into the LC-MS system for analysis.

The analytical conditions were as follows: column temperature, 35 °C; autosampler temperature, 15 °C; and flow rate, 0.3 mL/min. The gradient elution program was set as follows. Mobile phase A (0.1% formic acid) and mobile phase B (methanol) were set at 0 min, 15% B; 5 min, 70% B; and 6.5 min, 70% B. The ESI source was set in positive ionization mode; selected ion monitoring (SIM) mode (m/z 180 for phenacetin, m/z 152 for paracetamol and m/z 166 for IS-1) was used. The detector voltage was 1.5 kV, the heat block temperature was 200 °C; and the desolvation line temperature was 250 °C; nitrogen was used as nebulizing gas, with a flow rate of 1.5 L/min.

The calibration standard ranges used for phenacetin and paracetamol were 5–8000 μg/L and 10–8000 μg/L, respectively.

1.2 Untargeted metabolomics analysis

Serum and liver sample pretreatment, GC-MS and LC-MS analysis, data preprocessing, and metabolite identification were all based on our previous studies [1-3].

Sample pretreatment

For frozen liver samples, liver homogenates were prepared first. The same part of the left lobe of the liver from each rat was taken for tissue sample preparation. Ten volumes of pre-cold methanol were added to approximately 100 mg liver samples, followed by homogenization three times (5.5 m/s for 30 s) with 60 s intervals between each step. After two centrifugations (14,000 rpm, 4 °C, 10 min), the supernatant was removed for metabolomic analysis. Serum samples were thawed at room

temperature. For GC-MS analysis, 100 μL methanol was added to a 10 μL aliquot of serum or liver homogenate, and the mixture was thoroughly vortex-mixed for 15 min. After two centrifugations (14,000 rpm, 4 °C, 10 min), 80 μL supernatant was transferred to a brown glass vial, and then oximated with 25 μL MOX (10 mg/mL in pyridine) at 1200 rpm for 90 min at 37 °C. After vacuum drying (Labconco CentriVap, Kansas, MO, United States), the residue was silylated with 120 μL MSTFA:ethyl acetate (1:4, v/v) by incubating at 37 °C for 120 min, and then the supernatant was separated for GC-MS analysis.

For LC-MS analysis, 20 μL serum or 50 μL liver homogenate was thoroughly mixed with 140 μL or 100 μL acetonitrile, respectively. After a second centrifugation (4 °C, 14000 rpm, 10 min), the supernatant was separated for LC-MS analysis.

GC-MS analysis

GC-MS analysis was performed on GCMS-QP2010 Ultra (Shimadzu Inc., Kyoto, Japan) equipped with a Rtx-5MS capillary column (30 m × 0.25 mm ID, 0.25 μm, Restek, USA). Helium was employed as the carrier gas at a flow rate of 1mL/min. The oven temperature was initially set at 70 °C for 3 min, followed by an increase to 320 °C (10 °C/min), and maintained at 320 °C for 2 min. The temperature of the injector, transfer line, and ion source were set at 250, 250, and 200 °C, respectively. The mass spectrometer was operated in electron impact mode with the energy of 70 eV. Data acquisition was performed in full san mode with a 45–600 mass to charge ratio (m/z) range. A 1 μL sample was injected, with the split ratio of 50:1. GCMS solution version 2.7 (Shimadzu Inc., Kyoto, Japan) was used for spectra acquisition and data processing.

LC-MS analysis

LC-MS analysis was performed on an ultra-fast liquid chromatography (UFLC) system coupled with ion trap/time-of-flight hybrid mass spectrometry (IT/TOF-MS, Shimadzu Inc., Kyoto, Japan). Chromatographic separation was achieved by a Phenomenex Kinetex C18 column (100 × 2.1 mm, 2.6 μm, Phenomenex, United States). The column temperature was set at 40 °C. The gradient elution with 0.4 mL/min flow rate (phase A: 0.1% formic acid, phase B: acetonitrile) was carried out from 95% A to 5% A within 20 min and maintained at 5% A for 3 min. For mass analysis, ESI was set in both positive and negative ion mode with a 100–1000 m/z san range. The TOF analyzer detector voltage was 1.8 kV, and the interface voltage was set at 4.5 kV and -3.5 kV for

positive and negative mode, respectively. The curved desorption line and heat block temperature were both set at 200 °C. Nitrogen was used as the nebulizing gas, with a flow rate of 1.5 L/min. A 5 μL sample was injected for analysis. LCMS solution version 3.0 (Shimadzu Inc., Kyoto, Japan) was used for spectra acquisition and data processing.

Data preprocessing

Each chromatogram obtained from GC-MS and LC-MS analysis was processed for peak deconvolution and alignment using Profiling Solution version 1.1 (Shimadzu, Kyoto, Japan), followed by background-peak-filtering, 80% rule, limitation of QCs, missing data imputation, and normalization. The details of each step were as follows [3]:

(1) Background-peak filtering: each chromatogram was checked against the solvent blanks (inserted randomly in the analytical batch) to exclude possible sources of contamination, such as instrumental contamination or reagent impurities.

(2) 80% rule: retained the variables which were detectable in more than 80% samples in at least one group to minimize the effect of the missing values.

(3) QC sample limitation: removed the variables with RSD values higher than 30% in QC samples.

(4) Missing data imputation and normalization: replaced the missing values with a half of the minimum value found in the dataset. After the total area normalization for each sample, a resulting matrix was obtained and then prepared for further differential features screening and metabolite identification.

Metabolites identification

For GC-MS analysis, metabolites were preliminarily identified by a comparison of mass spectra and intensities with those available in National Institute of Standards and Technology (NIST 11) library. To minimize false discovery rates, only those peaks with similarity more than 75% were assigned for compound names and considered reliable. Some of the metabolites were further confirmed by standard compounds available in our lab.

For LC-MS analysis, the metabolites formulae were initially predicted by comparison of theoretical and observed m/z values and isotopic patterns using Formula Predictor in LCMS Solution software. Then the m/z values, formulae and the MS/MS fragmentations information were

compared with those provided by existing literature and online databases, such as HMDB (http://www.hmdb.ca/), METLIN (https://metlin.scripps.edu/), and Mass Bank (http://www.massbank.jp). To minimize false discovery rates, some of the metabolites were further confirmed by standard compounds available in our lab.

1.3 Quantification of BCAAs, Phe, and Tyr

A simple and rapid analytical method was developed for simultaneous quantification of Val, Leu, Ile, Phe, and Tyr based on our previous study with little modification [4].

For sample preparation, a total of 160 μL acetonitrile and 10 μL internal standard working solution (50 μg/mL $^{13}C_1$-leucine, IS-2) were added to 40 μL serum or liver homogenate and vortex-mixed for 5 min. After centrifugation (4 °C, 14,000 rpm, 10 min), an aliquot of 125 μL supernatant was transferred into another 1.5 mL Eppendorf tube and evaporated by vacuum drying at 37 °C. Subsequently, the residue was dissolved in 50 μL 1% formic acid. Finally, the reconstituted extract was centrifuged (4 °C, 14,000 rpm, 10 min) and the supernatant was separated for LC-MS/MS analysis.

For apparatus and analytical conditions, the linear gradient program was set as follows, with mobile phase A (0.1% formic acid) and mobile phase B (methanol): 0 min, 10% B; 1.5 min, 10% B; 3.5 min, 20% B; and 5 min, 30% B. The column was equilibrated for 6 min at 10% mobile phase B before each injection. The flow rate was 0.25 mL/min and the column temperature was kept at 40 °C. The injection volume was 2 μL with full loop injection. The mass spectrometer was operated in positive ion mode with multiple reaction monitoring (MRM). The optimal parameters were as follows: spray voltage, 4.5 kV; nebulizing gas (nitrogen), 3.0 L/min; drying gas (nitrogen), 15.0 L/min; desolvation line temperature, 250 °C; heat block temperature, 400 °C; and collision induced dissociation (CID) gas, 230 kPa. The main parameters for MS/MS detection of each analyte as well as the internal standard were summarized in Supplementary Table S1.

The calibration standard ranges used for biomarkers in serum and liver were 0.25-50 μg/mL and 2.5-500 μg/g, respectively.

References

1. Dai, D.; Tian, Y.; Guo, H.; Zhang, P.; Huang, Y.; Zhang, W.; Xu, F.; Zhang, Z. A pharmacometabonomic approach using predose serum metabolite profiles reveals differences

in lipid metabolism in survival and non-survival rats treated with lipopolysaccharide. *Metabolomics*. **2016**,*12*,2.
2. Zhang, P.; Chen, J.Q.; Huang, W.Q.; Li, W.; Huang, Y.; Zhang, Z.J.; Xu, F.G. Renal medulla is more sensitive to cisplatin than cortex revealed by untargeted mass spectrometry-based metabolomics in rats. *Sci Rep*. **2017**,*7*,44804.
3. Gao, Y.Q.; Li, W.; Chen, J.Q.; Wang, X.; Lv, Y.T.; Huang, Y.; Zhang, Z.J.; Xu, F.G. Pharmacometabolomic prediction of individual differences of gastrointestinal toxicity complicating myelosuppression in rats induced by irinotecan. *Acta Pharm Sin B*. **2019**,*9*,157-166.
4. Li, R.; Liu, P.; Liu, P.; Yuan, T.; Hua, Y.; Gao, Y.; Hua, H.; Chen, J.; Zhang, Z.; Yin, H. A novel liquid chromatography tandem mass spectrometry method for simultaneous determination of branched-chain amino acids and branched-chain α-keto acids in human plasma. *Amino Acids*. **2016**,*48*,1-10.

metabolites

2. Figures

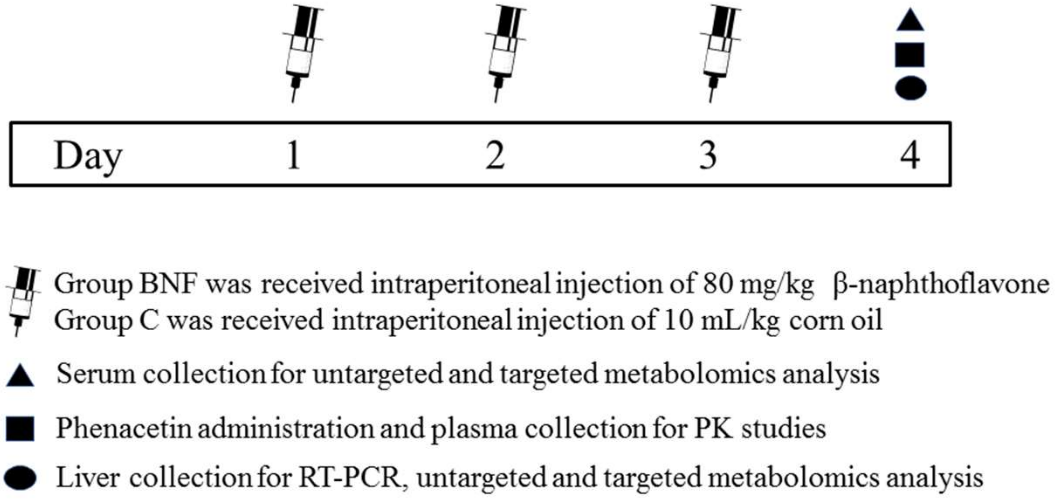

Figure S1. Experimental design.

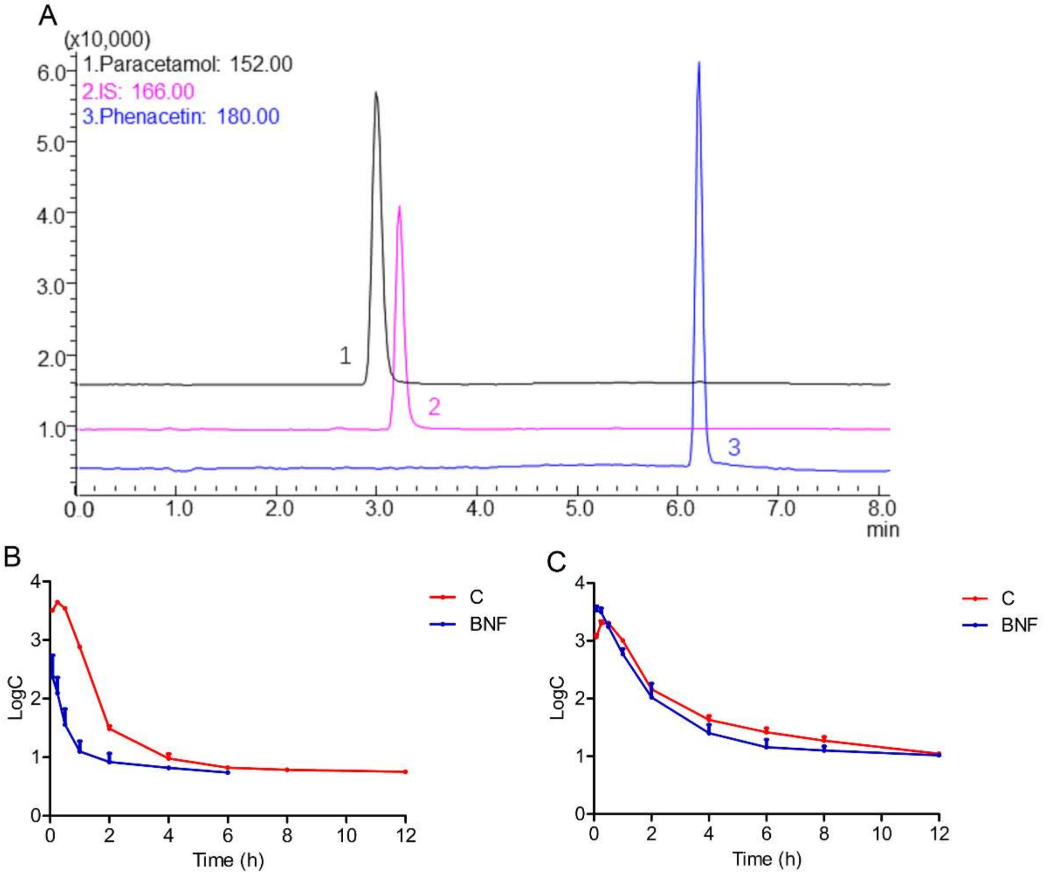

Figure S2. PK studies of phenacetin and paracetamol. (A) Representative LC-MS chromatograms of phenacetin, paracetamol, and IS (internal standard, pseudoephedrine hydrochloride) in plasma sample obtained at 1 h after dosed with phenacetin. (B) Concentration–time curve of phenacetin. (C) Concentration–time curve of paracetamol. Plasma concentration vs time curves are represented as a semi-log graph. The unit of concentration is μg/L. Data are expressed as mean ± SD, and n = 8 for each group. C: control group; BNF: β-naphthoflavone treatment group; LogC: logarithm of concentration.

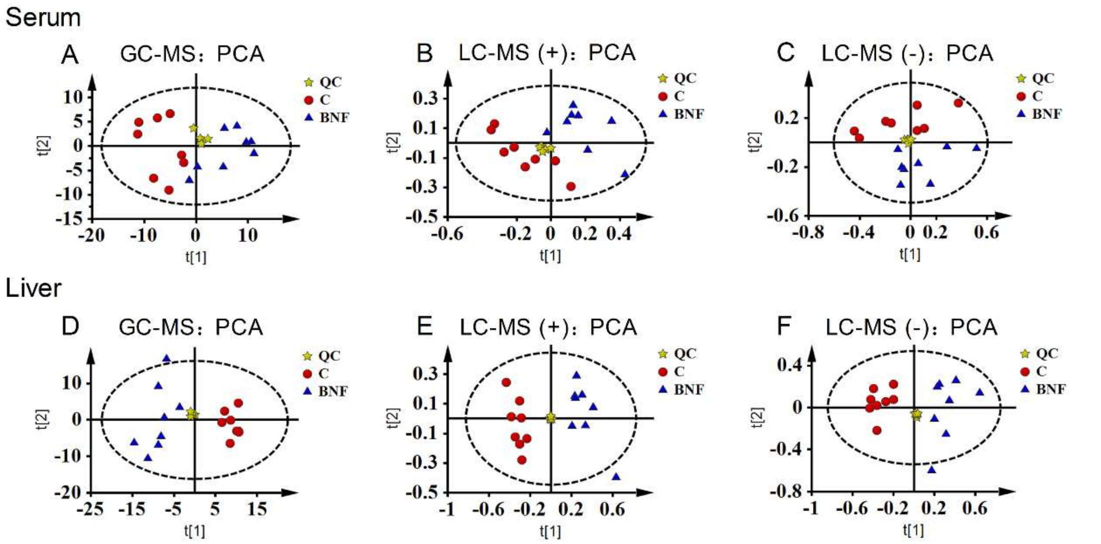

Figure S3. PCA score plots of the C group and BNF group in serum (A-C) and liver (D-F) samples, detected by GC-MS (A and D), LC-MS (+) (B and E), and LC-MS (-) (C and F) analysis. QC samples were clustered tightly in PCA score plots, and the BNF group was obviously separated from control group. C: control group; BNF: β-naphthoflavone treatment group; QC: quality control.

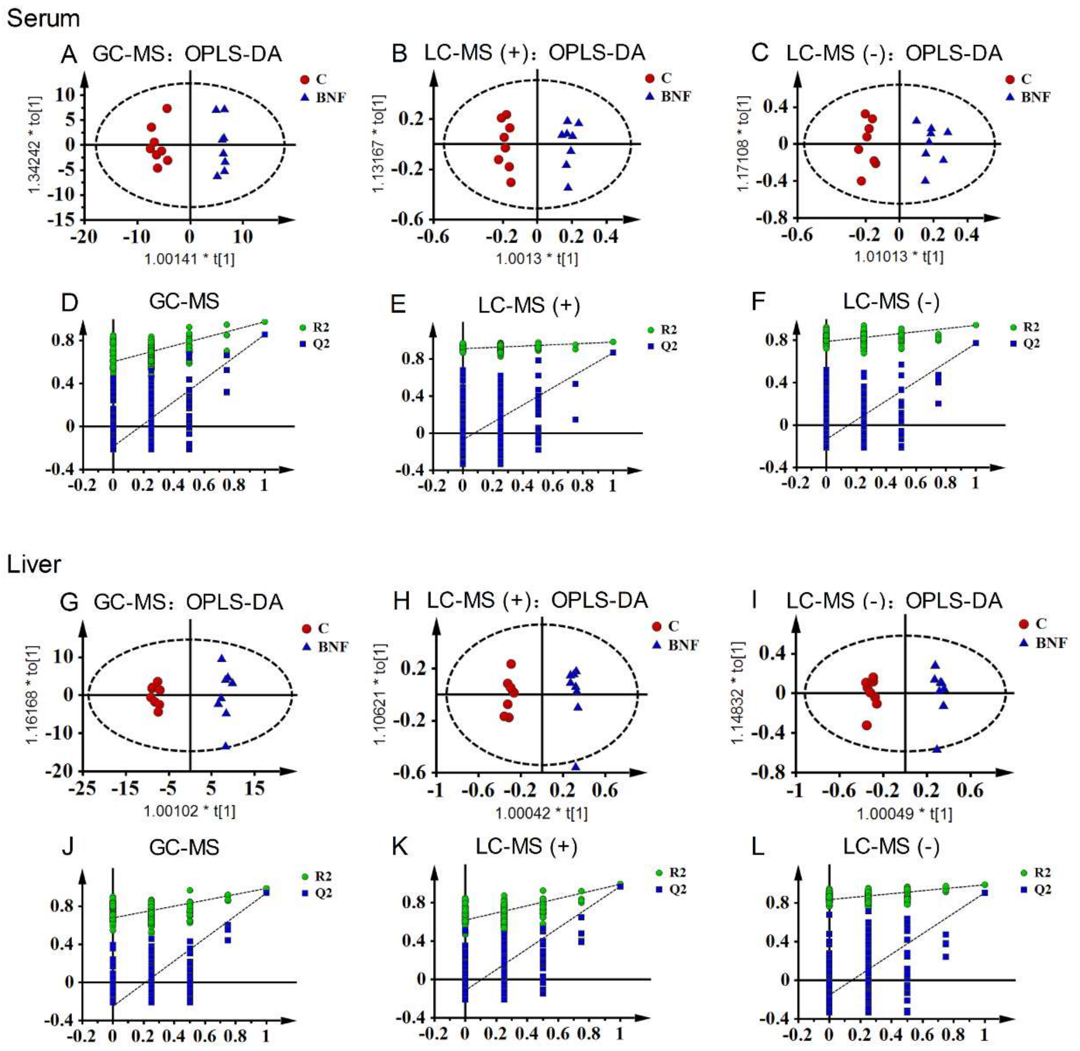

Figure S4. Screening differential metabolites between the C group and BNF group based on untargeted metabolomics analysis. OPLS-DA score plots (serum: A-C, liver: G-I) showed complete separation between the C and BNF group. The values of predictive ability parameter Q^2 are as follows: A, 0.863; B, 0.834; C, 0.838; G, 0.947; H, 0.957; I, 0.927. The permutation test results (200 times, serum: D–F, liver: J–L) of the corresponding PLS-DA models demonstrate that these OPLS-DA models had no overfitting.

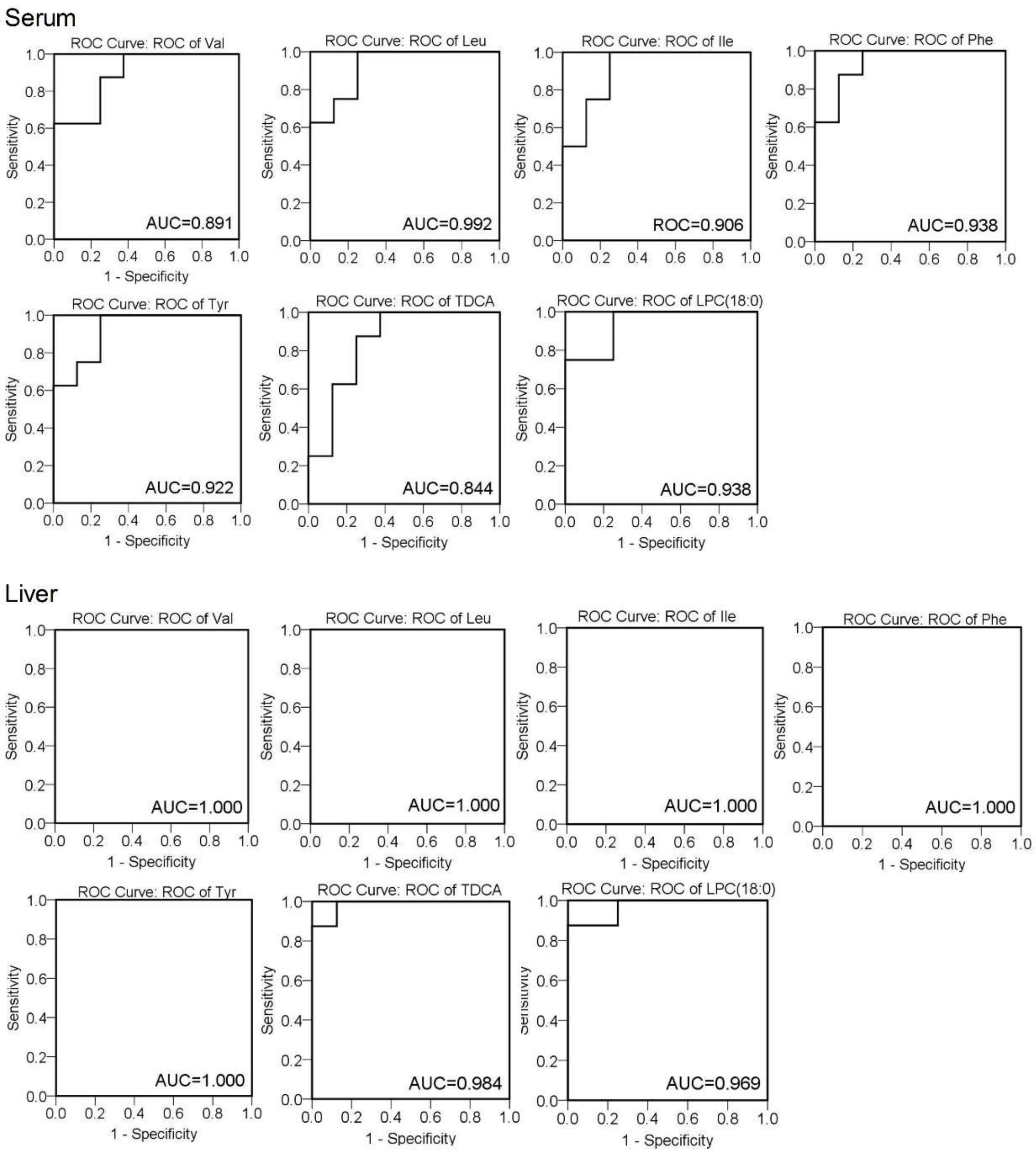

Figure S5. ROC curves of seven differential metabolites focalized based on untargeted metabolomics analysis of serum and liver.

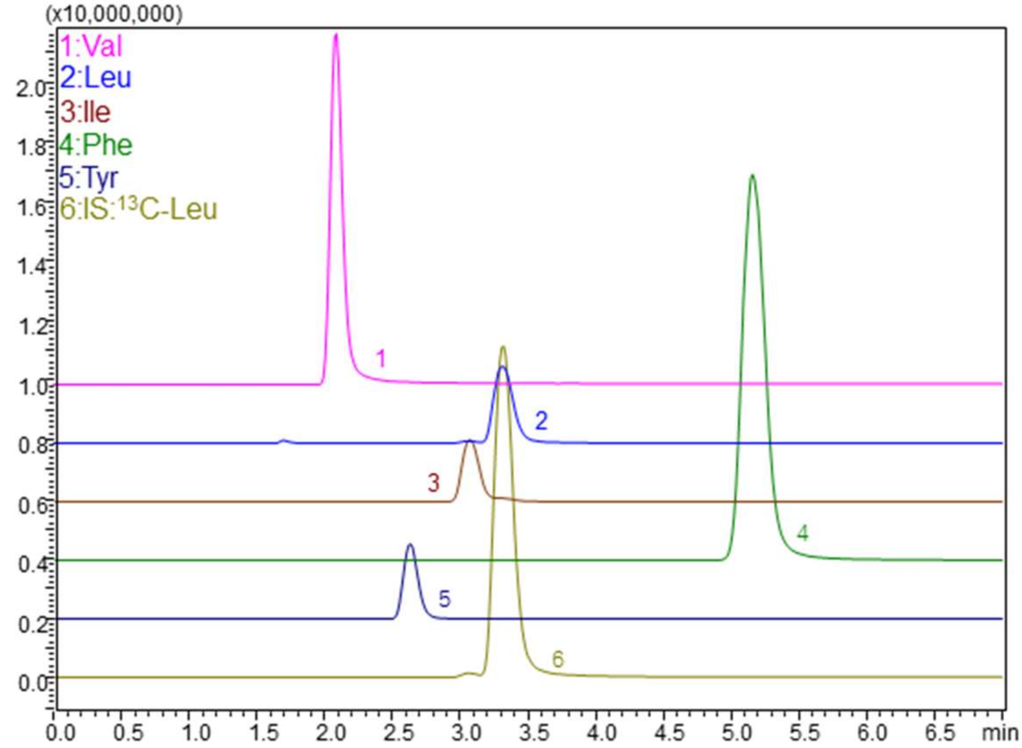

Figure S6. Representative MRM chromatograms of Val, Leu, Ile, Phe, Tyr, and IS (internal standard, $^{13}C_1$-leucine) in a serum sample of control rats.

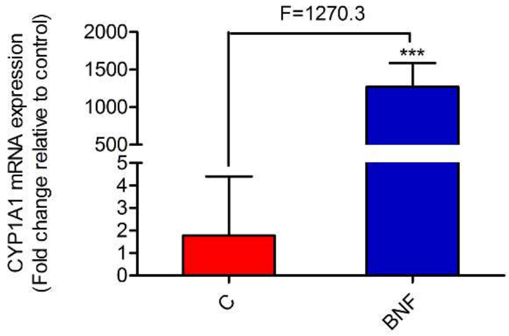

Figure S7. The relative expression of CYP1A1 mRNA after β-naphthoflavone administration. The determination of CYP1A1 mRNA level was performed according to Manuscript Materials Methods 2.4 and the sequences of the primers of *CYP1a1* and *β-actin* are summarized in Supplementary Table S2. The mRNA levels were normalized by *β-actin* expression and expressed as the fold change relative to control. Data are presented as mean ± SD, and n = 8 for each group. Unpaired Student's t-test. *** $p < 0.001$. C: control group; BNF: β-naphthoflavone treatment group; F: fold change.

3. Tables

Table S1. Main parameters for MS/MS detection of each analyte

Analyte	Molar Mass (g/mol)	Precusor ion (m/z)	Collision energy (V)	Product ion (m/z)
Val	117.15	118.1	-13	72.2
Leu	131.17	132.1	-25	43.1
Ile	131.17	132.1	-18	69.1
Phe	165.19	166.1	-14	120.2
Tyr	181.19	182.1	-27	91.0
IS（^{13}C-leucine）	132.17	133.1	-11	86.0

Table S2. Sequences of the primers of *CYP1a1* and *β-actin*

Gene	Sequence (5'-3')	Product size (bp)
CYP1a1	Forward: CATTGTGCCTGCCTCCTACTT<br>Reverse: GTTCCTGTGGGTCTCTGCTGT	81
β-actin	Forward: GGAGATTACTGCCCTGGCTCCTA<br>Reverse: GACTCATCGTACTCCTGCTTGCTG	150

Table S3. The changes of CYP1A2 mRNA level after β-naphthoflavone administration

	C group	BNF group
CYP1A2 mRNA level	0.46 ± 0.21	6.41 ± 1.80

CYP1A2 mRNA level was calculated using the $2^{-\Delta Ct}$ method and normalized by *β-actin* expression. Data are presented as mean ± SD, and *n* = 8 for each group. *Ct*: cycle threshold; △*Ct* = *Ct* of *CYP1a2* - *Ct* of *β-actin*; C: control group; BNF: β-naphthoflavone treatment group.

Table S4 List of differential metabolites in the serum of group C and BNF detected by GC-MS

NO.	Metabolites	RT (min)	Similarity	VIP value	*p*FDR	AUC-ROC	r value (metabolic ratio)[a]	r value (mRNA level)[b]	change trend[c]
1	Acetamide	6.235	90	1.14	0.016	0.875	0.63	0.54	↑
2	Phosphoric acid	8.335	82	1.33	0.003	0.953	0.62	0.57	↑
3	Valine	8.927	97	1.21	0.012	0.891	-0.63	-0.56	↓
4	Leucine	9.794	96	1.27	0.006	0.922	-0.66	-0.57	↓
5	Isoleucine	10.133	93	1.12	0.009	0.906	-0.68	-0.52	↓
6	Proline	13.405	90	1.21	0.003	0.953	-0.74	-0.66	↓
7	Pentanedioic acid	14.013	89	1.55	0.000	1.000	0.77	0.68	↑
8	Asparagine	15.213	87	1.32	0.005	0.938	-0.73	-0.65	↓
9	Tyrosine	18.231	92	1.26	0.006	0.922	-0.79	-0.65	↓
10	Octadecanoic acid	21.039	82	1.49	0.000	1.000	0.72	0.68	↑
11	Cystine	21.700	82	1.31	0.003	0.953	-0.90	-0.68	↓
12	C22:6	23.820	79	1.50	0.000	1.000	0.80	0.69	↑
13	Tocopherol	28.289	78	1.58	0.000	1.000	0.83	0.67	↑
14	Cholesterol	28.584	94	1.61	0.000	1.000	0.76	0.79	↑

C22:6: cis-4,7,10,13,16,19-docosahexaenoic acid

Table S5 List of differential metabolites in the serum of group C and BNF detected by LC-MS

NO.	Metabolites	m/z	RT (min)	+/-	Ion form	MS/MS fragments	VIP value	*p*FDR	AUC-ROC	r value (metabolic ratio)[a]	r value (mRNA level)[b]	change trend[c]
1	Phenylalanine	166.0868	1.13	+	[M+H] +	120.084	1.07	0.007	0.938	-0.77	-0.62	↓
2	LysoPE(16:0)	452.3515	13.063	-	[M-H]-	255.2753, 196.0677	2.23	0.012	0.891	0.52	0.59	↑
3	LysoPE(18:2)	476.3561	12.562	-	[M-H]-	279.277	3.27	0.004	0.969	0.77	0.70	↑
		478.2925	12.566	+	[M+H] +	460.2813, 337.2755						
4	LysoPE(18:0)	482.3241	14.607	+	[M+H] +	464.3123, 341.3015	2.26	0.007	0.969	0.75	0.65	↑
5	TDCA	498.3692	7.936	-	[M-H]-	498.3712, 497.3543	1.98	0.028	0.844	-0.74	-0.51	↓
6	LysoPE(20:4)	500.359	12.601	-	[M-H]-	303.2823, 259.2831	3.26	0.005	0.938	0.64	0.61	↑
		502.2945	12.607	+	[M+H] +	484.2823, 361.2713						
7	LysoPE(18:1)	478.3725	13.466	-	[M-H]-	281.2895, 253.2584	1.76	0.003	0.953	0.81	0.79	↑
8	LysoPC(18:1)	566.4373	13.795	-	[M+HCOO]-	506.4084, 417.3253	5.01	0.009	0.906	0.71	0.68	↑
		522.3552	13.804	+	[M+H] +	504.3431, 445.2723						
9	LysoPC(20:1)	550.3864	15.461	+	[M+H] +	532.3479, 184.0675	1.08	0.026	0.844	0.55	0.66	↑
10	LysoPC(22:6)	568.3385	12.796	+	[M+H] +	550.3311, 184.0712	2.57	0.000	1.000	0.57	0.70	↑
		612.4294	12.796	-	[M+HCOO]-	552.3943, 283.2881						
11	LysoPC(18:0)	568.4455	13.795	-	[M+HCOO]-	508.4233, 283.3085	1.58	0.005	0.938	0.58	0.69	↑
12	LysoPC(22:5)	570.3556	13.228	+	[M+H] +	552.3482, 184.0693	3.12	0.000	1.000	0.81	0.66	↑
13	LysoPC(22:4)	572.3708	14.039	+	[M+H] +	554.3585, 184.0731	2.33	0.000	1.000	0.72	0.68	↑

TDCA: taurodeoxycholic acid

Table S6 List of differential metabolites in the liver of group C and BNF detected by GC-MS

NO.	Metabolites	RT (min)	Similarity	VIP value	*p*FDR	AUC-ROC	r value (metabolic ratio)[a]	r value (mRNA level)[b]	change trend[c]
1	Ethanolamine	5.673	98	1.54	0.000	1.000	-0.63	-0.72	↓
2	Aminobutyric acid	8.205	92	1.33	0.003	0.953	0.61	0.66	↑
3	Phosphoric acid	8.319	87	1.40	0.003	0.969	-0.70	-0.73	↓
4	Valine	8.921	97	1.57	0.000	1.000	-0.75	-0.64	↓
5	Leucine	9.794	96	1.60	0.000	1.000	-0.76	-0.68	↓
6	Isoleucine	10.135	92	1.60	0.000	1.000	-0.74	-0.71	↓
7	Aspartic acid	13.308	91	1.42	0.002	0.969	-0.76	-0.69	↓
8	Ornithine	14.495	76	1.34	0.002	0.969	-0.77	-0.74	↓
9	Phenylalanine	14.702	92	1.52	0.000	1.000	-0.78	-0.71	↓
10	9H-Purine	16.813	93	1.40	0.002	0.969	-0.75	-0.74	↓
11	Tyrosine	18.221	76	1.55	0.000	1.000	-0.68	-0.71	↓
12	7H-purine	19.116	96	1.14	0.000	0.984	-0.74	-0.82	↓
13	Hexadecanoic acid	19.136	84	1.33	0.002	0.969	-0.73	-0.82	↓
14	Uric acid	19.953	76	1.50	0.000	1.000	-0.71	-0.78	↓
15	Uridine	20.298	79	1.37	0.000	0.984	-0.69	-0.76	↓
16	Oleic acid	20.794	88	1.52	0.002	0.969	-0.66	-0.72	↓
17	Tryptophan	20.968	79	1.59	0.000	0.984	-0.75	-0.68	↓

metabolites

Table S7 List of differential metabolites in the liver of group C and BNF detected by LC-MS

NO.	Metabolites	m/z	RT (min)	+/-	Ion form	MS/MS fragments	VIP value	*p*FDR	AUC-ROC	r value (metabolic ratio)[a]	r value (mRNA level)[b]	change trend[c]
1	Hypoxanthine	137.0451	0.653	+	[M+H] +	109.1611, 92.1871	1.20	0.003	0.938	-0.70	-0.62	↓
2	Carnitine	162.1112	0.655	+	[M+H] +	163.0973, 103.0393	1.72	0.000	1.000	0.64	0.61	↑
3	Acetylcarnitine	204.1179	0.658	+	[M+H] +	145.047	2.36	0.000	1.000	0.67	0.74	↑
4	Glutathione	308.0888	0.64	+	[M+H] +	179.0497, 162.0234	2.97	0.002	0.953	0.73	0.67	↑
5	GDCA	450.3172	9.018	+	[M+H] +	432.3122, 414.3014	1.40	0.000	1.000	-0.82	-0.80	↓
6	GCA	464.3762	7.996	-	[M-H]-	463.364	1.48	0.030	0.828	-0.54	-0.55	↓
7	LysoPE(18:2)	478.2927	12.335	+	[M+H] +	337.2723, 281.2938	2.06	0.000	0.984	-0.69	-0.72	↓
		476.3559	12.331	-	[M-H]-	279.2771, 214.0734						
8	TMCA	514.3669	7.932	-	[M-H]-	496.4564, 353.1872	6.32	0.000	1.000	0.89	0.79	↑
9	TDCA	498.3699	7.952	-	[M-H]-	482.2939, 464.2843	4.56	0.000	0.984	-0.77	-0.77	↓
10	LysoPE(20:4)	500.3577	12.398	-	[M-H]-	303.2341, 259.1584	2.51	0.008	0.906	-0.59	-0.63	↓
		502.2934	12.401	+	[M+H] +	484.2842, 361.2738						
11	TCA	516.299	7.939	+	[M+H] +	464.3113, 446.2935	1.88	0.000	0.984	0.83	0.80	↑
12	LysoPC(18:0)	524.3713	14.802	+	[M+H] +	506.3633, 184.0732	1.19	0.001	0.969	0.75	0.69	↑
13	LysoPE(22:6)	524.3621	12.421	-	[M-H]-	327.2861, 283.2881	2.16	0.009	0.891	-0.65	-0.53	↓
		526.292	12.427	+	[M+H] +	508.2842, 385.2737						
14	LysoPC(20:4)	544.3397	12.625	+	[M+H] +	526.3081, 184.0754	2.78	0.001	0.953	-0.57	-0.64	↓
		588.4255	12.619	-	[M+HCOO]-	530.2217, 303.6712						
15	PI(20:4)	619.3893	11.933	-	[M-H]-	303.282	1.70	0.005	0.922	-0.59	-0.64	↓

GDCA: glycodeoxycholic acid; GCA: glycocholic acid; TMCA: tauromuricholic acid; TDCA: taurodeoxycholic acid; TCA: taurocholic acid; LysoPC: lysophosphatidylcholine; LysoPE, lysophosphatidylethanolamine; PI: phosphoinositol.

[a]Correlation coefficients of Spearman correlation analysis between differential metabolites and metabolic ratio. [b]Correlation coefficients of Spearman correlation analysis between differential metabolites and mRNA level of CYP1A2. [c]Change trends of differential metabolites based on area normalization data in untargeted metabolomics. ↓ decreasing change trend after β-naphthoflavone administration. ↑ increasing change trend after β-naphthoflavone administration. The value of the metabolic ratio reflected the activity of CYP1A2, and a higher value represented a higher activity. CYP1A2 mRNA expression was calculated using the $2^{-\Delta Ct}$ method. *Ct*: cycle threshold; $\triangle Ct = Ct$ of *CYP1a2* - *Ct* of *β-actin*.

Table S8 BCAAs, Phe, and Tyr concentrations in the serum (μg/mL) and liver (μg/g)

	Serum		Liver	
	C group	BNF group	C group	BNF group
Val	15.92 ± 2.45	11.12 ± 1.01	78.00 ± 6.56	49.56 ± 6.12
Leu	16.87 ± 2.24	11.95 ± 1.34	80.50 ± 7.12	49.42 ± 5.79
Ile	8.73 ± 1.28	6.21 ± 0.88	35.79 ± 2.67	22.54 ± 2.74
Phe	16.93 ± 1.88	13.95 ± 0.78	60.55 ± 6.86	37.09 ± 7.00
Tyr	11.17 ± 1.78	7.78 ± 0.89	57.19 ± 6.23	32.42 ± 6.64

Data are presented as mean ± SD; *n* = 8 for each group. C: control group; BNF: β-naphthoflavone treatment group.

Table S9 The ratio of Phe to Tyr concentration in the serum and liver

	Serum		Liver	
	C group	BNF group	C group	BNF group
Phe/Tyr	1.53 ± 0.14	1.81 ± 0.18	1.06 ± 0.08	1.17 ± 0.20

Data are presented as mean ± SD; *n* = 8 for each group. C: control group; BNF: β-naphthoflavone treatment group.
